# Designer rhamnolipids by reduction of congener diversity: production and characterization

**DOI:** 10.1186/s12934-017-0838-y

**Published:** 2017-12-14

**Authors:** Till Tiso, Rabea Zauter, Hannah Tulke, Bernd Leuchtle, Wing-Jin Li, Beate Behrens, Andreas Wittgens, Frank Rosenau, Heiko Hayen, Lars Mathias Blank

**Affiliations:** 1 iAMB-Institute of Applied Microbiology, ABBt-Aachen Biology and Biotechnology, RWTH Aachen University, Worringerweg 1, 52074 Aachen, Germany; 2 Institute of Inorganic and Analytical Chemistry, University of Münster, Corrensstraße 30, 48149 Münster, Germany; 3 Present Address: Doehler GmbH, Riedstraße 7-9, 64295 Darmstadt, Germany; 4 Ulm Center for Peptide Pharmaceuticals (U-PEP), Ulm-University, Albert-Einstein-Allee 11, 89081 Ulm, Germany

**Keywords:** Rhamnolipid, Non-pathogenic *Pseudomonas*, Hydroxyalkanoyloxy alkanoate, Designer biosurfactants, Bioeconomy

## Abstract

**Background:**

Rhamnolipids are biosurfactants featuring surface-active properties that render them suitable for a broad range of industrial applications. These properties include their emulsification and foaming capacity, critical micelle concentration, and ability to lower surface tension. Further, aspects like biocompatibility and environmental friendliness are becoming increasingly important. Rhamnolipids are mainly produced by pathogenic bacteria like *Pseudomonas aeruginosa*. We previously designed and constructed a recombinant *Pseudomonas putida* KT2440, which synthesizes rhamnolipids by decoupling production from host-intrinsic regulations and cell growth.

**Results:**

Here, the molecular structure of the rhamnolipids, i.e., different congeners produced by engineered *P. putida* are reported. Natural rhamnolipid producers can synthesize mono- and di-rhamnolipids, containing one or two rhamnose molecules, respectively. Of each type of rhamnolipid four main congeners are produced, deviating in the chain lengths of the β-hydroxy-fatty acids. The resulting eight main rhamnolipid congeners with variable numbers of hydrophobic/hydrophilic residues and their mixtures feature different physico-chemical properties that might lead to diverse applications. We engineered a microbial cell factory to specifically produce three different biosurfactant mixtures: a mixture of di- and mono-rhamnolipids, mono-rhamnolipids only, and hydroxyalkanoyloxy alkanoates, the precursors of rhamnolipid synthesis, consisting only of β-hydroxy-fatty acids. To support the possibility of second generation biosurfactant production with our engineered microbial cell factory, we demonstrate rhamnolipid production from sustainable carbon sources, including glycerol and xylose. A simple purification procedure resulted in biosurfactants with purities of up to 90%. Finally, through determination of properties specific for surface active compounds, we were able to show that the different mixtures indeed feature different physico-chemical characteristics.

**Conclusions:**

The approach demonstrated here is a first step towards the production of designer biosurfactants, tailor-made for specific applications by purposely adjusting the congener composition of the mixtures. Not only were we able to genetically engineer our cell factory to produce specific biosurfactant mixtures, but we also showed that the products are suited for different applications. These designer biosurfactants can be produced as part of a biorefinery from second generation carbon sources such as xylose.

**Electronic supplementary material:**

The online version of this article (10.1186/s12934-017-0838-y) contains supplementary material, which is available to authorized users.

## Background

Rhamnolipids are biosurfactants that can be utilized for a wide range of applications. Their amphiphilic characteristics makes them suited for example as emulsifying [1] and foaming agents [2]. Furthermore, they are biodegradable and therefore have a low impact on the environment [3]. Numerous publications about rhamnolipid-producing microorganisms exist. Many of these lack proper characterization of the product and/or the producing strain itself [4].

### Applications

Rhamnolipids already find application in a variety of industries with even more potential uses. For example, their properties render them especially suited for environmental applications, such as bioremediation of hydrocarbons, organic pollutants, and heavy-metal-contaminated sites, enhanced oil recovery, and treatment of oil spills [5]. Rhamnolipids are excellent suspending agents, facilitating the superior break-down of pollutants compared to many conventional synthetic surfactants. Consequently, lower amounts of surface active molecules have to be introduced into the polluted areas [6], adding up to high biocompatibility. Apart from treatment of polluted areas on the ground, rhamnolipids can also be used to treat marine oil spills [3].

Rhamnolipids already have a strong foothold in the chemical, cosmetic, pharmaceutical, and food industries [7] and are the only biosurfactants approved for use in the latter three [8]. In cosmetics, their advantages over synthetic biosurfactants include low irritancy and high skin compatibility [9]. This can explain why they are already used in Japan as cosmetic additives [10] with over 100 patents filed as of 2016 for their cosmetic use [11], including in liposomes and emulsions production [10]. Several properties make rhamnolipids interesting for the pharmaceutical industry [12], including enhancement of burn wound healing [13] as well as antimicrobial activity, and the stimulation of the immune system of animals [14]. They also find use as environmental friendly cleaning agents [15] with over 150 related patents filed [11]. In food production, biosurfactants are used generally to control consistency, delay staling, to solubilize flavor oils in bread and ice cream products [6], and are used as fat stabilizers and antispattering agents during cooking [16]. Rhamnolipids specifically have been shown to enhance dough stability and texture and conservation of bakery products [6].

A completely different field of application is crop science. Rhamnolipids were suggested to play a role in mediating resistance of plants against microbes and to stimulate the plant immune system [14]. Furthermore, rhamnolipids from rhizosphere microorganisms can play important roles, for example by reducing the damping-off disease in plants [17, 18].

Given these wide-ranging applications of rhamnolipid biosurfactants, optimum physico-chemical properties might be variable and these are tuned by different molecular structures, briefly summarized in the next paragraph.

### Structure and properties

**Fig. 1:**
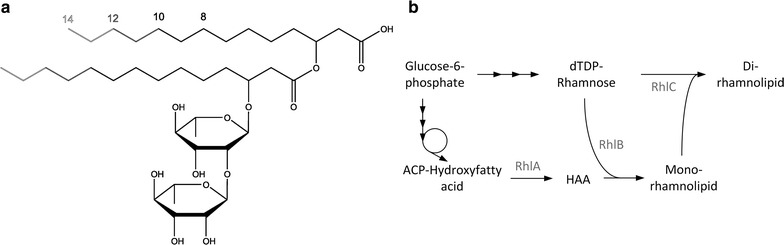
**a** Structure of rhamnolipids. The upper part of the molecule is formed by the hydrophobic moiety, the hydroxyalkanoyloxy alkanoate (HAA). The chain lengths of the β-hydroxy-fatty acids in this dimer can vary. One or two rhamnose molecules are bound by a glycosidic bond. The sugar molecules are the hydrophilic moiety of the molecule. Molecules with one rhamnose are called mono-rhamnolipids, while the here depicted molecule with two rhamnoses is a di-rhamnolipid. **b** Biosynthesis pathways of rhamnolipids. Based on glucose two pathways are required for rhamnolipid synthesis. In the lower part, activated β-hydroxy-fatty acids are formed via fatty acid de novo synthesis, which are fused by the enzyme RhlA. In the upper part, activated rhamnose is synthesized and subsequently coupled to the β-hydroxy-fatty acid dimer by the rhamnosyltransferase I (RhlB). The rhamnosyltransferase II (RhlC) finally adds another sugar molecule to yield a di-rhamnolipid. Enzyme names are printed in grey

Rhamnolipids are a diverse group of molecules with more than 60 reported congeners [19]. The amphiphilic part of rhamnolipids is formed by the hydrophilic sugar rhamnose and the hydrophobic moiety (Fig. 1), composed of a dimer of two esterified β-hydroxy-fatty acids. Overall structural differences result from variations in the sugar and in the hydrophobic moiety. The number of either the sugar residues or the β-hydroxy-fatty acids is usually either one or two, but up to three β-hydroxy-fatty acids were reported [20]. On the other hand, the number of carbon atoms in the β-hydroxy-fatty acids can vary widely between six [21] and 24 [22], with most scientific papers reporting values between eight and 16 [19].

All rhamnolipid producing cell factories have nevertheless one common trait: they produce mixtures regarding the number of rhamnose residues as well as the length of the β-hydroxy-fatty acid chains and usually one congener is predominant. The most commonly used bacterium for rhamnolipid production, *Pseudomonas* *aeruginosa*, for example produces mainly the Rha-Rha-C_10_-C_10_ congener. This term refers to a di-rhamnolipid with two rhamnoses (Rha) and two β-hydroxy-fatty acids, each with ten carbon atoms (C_10_). Furthermore, it produces three minor congeners in significant amounts containing C_8_ and C_12_ β-hydroxy-fatty acids and a C_12_ β-hydroxy-fatty acid with one unsaturation. All these four congeners also exist with only one rhamnose residue (e.g., Rha-C_10_-C_10_). Thus, a total of eight main rhamnolipid congeners are produced by the rhamnolipid synthesis pathway of *P. aeruginosa* [23].

In fermentations with *P. aeruginosa* di-rhamnolipids are the predominant biosurfactants produced, while other microorganisms mostly produce mono-rhamnolipids [24, 25]. Nevertheless, most microorganisms mainly synthesize di-rhamnolipids [26–30]. The number of rhamnose molecules forming the hydrophilic moiety has a decisive effect on the biosurfactant’s surface-active properties like critical micelle concentrations [5], which in turn determines the suitability for different applications. The adjustment of properties can be achieved by specifically synthesizing only single congeners or particular mixtures out of the whole product spectrum. This versatility based on the structural diversity of the molecule, offers great possibilities for tailor-made designer rhamnolipids.

Prior to engineering a microbial cell factory for designer rhamnolipid production, comprehensive insight into their biosynthesis is required. However, a poor understanding of intrinsic secondary metabolite regulatory cascades currently limits the ability to effectively manipulate the wild-type producer genetic background.

### Biosynthesis of rhamnolipids

The rhamnolipid pathway in *P. aeruginosa* comprises three key enzymes and is based on the two precursors rhamnose and β-hydroxy-fatty acid (Fig. 1). The activated β-hydroxy-fatty acid hydroxyacyl-ACP is generated in the fatty acid de novo synthesis. Subsequently the first rhamnolipid specific enzyme 3-hydroxyacyl-ACP:3-hydroxyacyl-ACP *O*-3-hydroxy-acyl-transferase (RhlA) connects two hydroxyacyl-ACP molecules to form a dimer called hydroxyalkanoyloxy alkanoate (HAA). This molecule does not contain a rhamnose unit and thus is not a rhamnolipid. Due to its ester, carboxyl, and hydroxy groups and resulting amphiphilic structure, it nevertheless is a biosurfactant. The second precursor originates in six reactions from glucose. Activated dTDP-l-rhamnose is then fused by rhamnosyltransferase I (RhlB) to the HAA molecule to yield a mono-rhamnolipid. The second rhamnosyltransferase (RhlC) adds a second sugar to the mono-rhamnolipid, finally leading to the di-rhamnolipid biosurfactant.

The environmental impact of rhamnolipid biosurfactants includes the source of carbon used in biosynthesis. Native rhamnolipid producers mainly use hydrophobic carbon sources [31]. Recombinant systems enable the use of more sustainable carbon sources. Sustainable substrates include xylose from, for example, corn or wheat straw, which do not compete with food production. Crude glycerol is also suitable because it is a waste stream from biodiesel production plants and is depending on the region of low cost and available at high quantities. Rhamnolipid production with glycerol as carbon source has already been shown with the native producer *P. aeruginosa* [32]. Glucose is the predominant substrate for biotechnological research applications and via starch hydrolysis often used in industry [33]. Due to the high price of purified sugars the use as substrate is in general not possible for the production of low price bulk chemicals such as a biosurfactants.

In this work, we report genetically engineered cell factories that synthesize specific rhamnolipid biosurfactant mixtures from renewable resources such as xylose. This is achieved via the tailored expression of rhamnolipid synthesis genes from *P. aeruginosa* PA01 in recombinant *Pseudomonas putida* cell factories. One mixture contains only mono-rhamnolipid species (four congeners). A second biosurfactant mixture contains mono- and di-rhamnolipids (eight congeners in total) and the third product is a mixture of four congeners of a biosurfactant composed only of the β-hydroxy-fatty acids of the rhamnolipid molecule, the HAA. We also present a simple purification procedure for all three biosurfactants. Finally, we were able to determine properties relevant for biosurfactants. This study also contributes to the growing literature on the production of secondary metabolites by recombinant *P. putida*. An excellent review on this topic was recently published [34]. The results are discussed in the context of production and applications of designer rhamnolipids.

## Methods

### Bacterial strains, culture conditions, and plasmids

The used bacterial strains *P. putida* KT2440 [35, 36], *Pseudomonas taiwanensis* VLB120 (formerly known as *Pseudomonas* sp. strain VLB120) [37], and *E. coli* DH5α [38] were routinely cultivated in LB medium (10 g/L tryptone, 5 g/L yeast extract, 10 g/L NaCl). *E. coli* was cultivated at 37 °C, while *P. putida* and *P.* *taiwanensis* were grown at 30 °C. Bacteria containing derivatives of vector pBBR1 were selected by adding 10 µg/mL tetracycline to LB-agar and liquid cultures. For selecting pSEVA241 derivatives kanamycin with a concentration of 50 µg/mL was added.

### Construction of plasmids

Plasmid pPS05 (for mono-rhamnolipid production) was previously constructed [39]. pWJ02 (for di-rhamnolipid synthesis) is a derivative of pPS05. The additional Gen *rhlC* was taken from a previously constructed plasmid carrying only *rhlC* (pVLT33_*rhlC*). Using PCR primers PS13 and PS14 *rhlC* was amplified and placed under the control of the synthetic promoter no. 16 (SynPro16) and an artificial RBS. With restriction enzyme *Asc*I the fragment and the plasmid pPS05 were cut and subsequently ligated.

pSB01 (for HAA production) was constructed by amplifying *rhlA* from a plasmid previously constructed that was already equipped with the engineered RBS and the synthetic promoter no. 8 (SynPro8). As a backbone, pSEVA241 was used. Both vector and fragment were cut with restriction enzymes *Kpn*I and *Sph*I and subsequently ligated.

Plasmid pVLT33_*rhlABC* (also for di-rhamnolipid synthesis) was constructed previously [40].

### Biosurfactant production

Biosurfactant production with recombinant pseudomonads was carried out using LB medium complemented with 10 g/L glucose and the respective antibiotic. The bacteria were cultivated in 500 mL shake flasks without baffles filled with 10% of their nominal volume. The experiments were executed in a Multitron shaker by Infors AG (Bottmingen, Switzerland). The temperature was maintained at 30 °C and flasks were shaken at 250 rpm with a shaking diameter of 25 mm. The humidity was controlled and kept at 80%.

When carbon sources other than glucose were utilized, the available moles of carbon were kept roughly constant. Thus, xylose (C_5_) and glucose (C_6_) were added at 10 g/L, while 20 g/L of glycerol (C_3_) was supplied.

Higher scale production was performed in Fernbach shake flasks filled with 500 mL LB medium, 10 g/L glucose, and the respective antibiotic (as described above). Flasks were cultivated in a Multitron shaker by Infors AG (Bottmingen, Switzerland) at 50 mm shaking diameter and 200 rpm. Glucose was added when its concentration fell below 1 g/L (at 38, 52, 91, and 140 h).

### Biosurfactant quantification

RP-HPLC-CAD was used for rhamnolipid quantification similarly to the previously published method [41]. Briefly a reversed phase chromatography (C18 column) coupled to a corona charged aerosol detector was used. The detailed procedure is described in Additional file 1: Section 1.3.

### Identification of rhamnolipid congeners

Verification of the chromatographic peak assignments of the RP-HPLC-CAD method was carried out by HPLC coupled to tandem mass spectrometry (LC–MS/MS) as described in [42]. Briefly, an Alliance 2695 separations module coupled to a Micromass Quattro micro triple quadrupole mass spectrometer (both Waters Corporation, Milford, MA, USA) was used. Full scan mass spectrometric detection in the range *m/z* 100–1000 was carried out in ESI-negative mode. Structural information was provided by additional MS/MS experiments (product ion scans).

### Biosurfactant purification

The cells were first separated from the culture broth by centrifugation at 13,000 rpm for 10 min in a Sorvall RC 5B Plus centrifuge from Thermo Fisher Scientific Inc. (Waltham, MA, USA) using 250 mL steel bottles. Subsequently, the supernatant was mixed with acetone at a ratio of 1:1 to precipitate remaining dissolved proteins. After stirring the mixture for about 1 h, the whole broth was again centrifuged under the same conditions. Afterwards the acetone was evaporated (in a water bath at 75 °C and under a constant flow of compressed air). When the acetone was completely evaporated, a final centrifugation step was performed (at the same conditions as stated above). The supernatant was then filtered using a Supor^®^-200 0.2 µm, 142 mm filter from Pall (Ann Arbor, MI, USA). Finally, the pH of the filtrate was increased to 10 with a 2 M NaOH solution.

The prepared supernatant was pumped onto an LC glass column with a length of 64 cm and an inner diameter of 2.44 cm (LATEK Labortechnik-Geräte GmbH, Eppelheim, Germany). The bed consisted of Europrep II 60-60 C18H from Knauer (Berlin, Germany) with a height of 20 cm. The used pump was a System Gold 125 Solvent Module from Beckmann Coulter (Krefeld, Germany). The column was primed with ethanol for about 15 min with 2–6 bed volumes per hour (according to Küpper et al. [43]) and afterwards washed with water at a pH of 10. The filtrate was pumped onto the column with a flow rate of 2–5 mL/min. Next, the column was again flushed with water (until no more color was eluted from the column). To elute the biosurfactants an ethanol gradient with the following program was used: 30 min 30% EtOH, 30 min 50% EtOH, 30 min 75% EtOH, 10 min 95% EtOH, 30 min 100% EtOH. The flow rate was set to 0.5 bed volumes per hour. During the elution, the eluate was collected in fractions, each with 0.1 bed volumes. The biosurfactant concentrations were measured via HPLC-CAD as stated above. Finally, the column was again flushed with water (neutral pH). Purification of rhamnolipids by adsorption/desorption has already been shown [44].

The pH of each fraction was measured and increased to 10, if necessary. Subsequently, the solvents were evaporated in the rotary dryer SCANSPEED Scan Speed 40 connected to the SCANVAC Cooling Trap (LaboGene, Lynge, Denmark) and the Chemistry Hybrid Pump RC 6 by VACUUBRAND GmbH (Wertheim, Germany). The purity of each fraction was determined by weighing the solid residue and HPLC-CAD measurements (see “Determination of purity”).

#### Pyoverdine content

The pyoverdine content in the fractions was determined by measuring the fluorescence. To this end, a sample of the fractions from the elution was diluted 1:1 with water and 200 µL were filled into black 96 well plates Microfluor from Thermo Fisher Scientific Inc. (Waltham, MA, USA). Using the well plate reader Synergy Mx by BioTek (Bad Friedrichshall, Germany) the emission was measured at 470 nm after excitation at 400 nm.

#### Other impurities

The overall purity could be roughly assessed by observing the coloring of the samples. Dark brown samples were considered impure, while colorless samples were regarded as mainly free of impurities when the HPLC-CAD measurement detected no additional peaks. The color was also measured by absorption at 400 nm, which was carried out in a Cary 60 UV–Vis spectrophotometer by Agilent (Santa Clara, CA, USA).

#### Determination of purity

The fractions were dried until they were completely free of water. 1 mg was taken from the sample and dissolved in 50% acetonitrile. In this sample the biosurfactant concentration was measured by HPLC-CAD. The determined amount was compared with the weighed amount to calculate the purity of the product.

### Property determination

The determination of different properties of HAAs, mono-rhamnolipids, and di-rhamnolipids was carried out with the aid of five different tests. Each test was performed as triplicate. We investigated the following properties: foam formation, emulsion stability, antifoam effectiveness, coagulation, and critical micelle concentration. The detailed description is presented in Additional file 1: Section 1.4.

To obtain the sample material, suitable fractions from the purification were mixed achieving representative congener content. The biosurfactants used to test the properties had a purity of 90% for the HAAs, 80% for the mono-rhamnolipids, and 65% for the di-rhamnolipids. All tests were also carried out with sodium dodecyl sulfate (SDS), which is a commonly used synthetic surfactant and which served here as reference.

## Results

### Biosurfactant production

For the production of the different biosurfactant mixtures, plasmids carrying the respective genes were constructed and introduced into *Pseudomonas* strains. All experiments were carried out in shake flasks followed by a first scale-up by increasing flask volumes.

#### Mono-rhamnolipid production

**Fig. 2:**
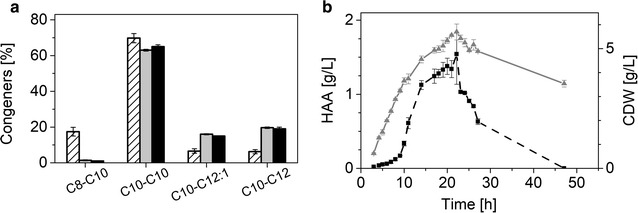
**a** Surfactant congeners produced by the different microbial cell factories. The striped columns represent the congener distribution for the HAAs produced by *P. taiwanensis* VLB120, the grey columns show the congener for the mono-rhamnolipids, and the black columns depict di-rhamnolipid congeners. **b** HAA production with recombinant *P. putida* KT2440 pSB01. The courses of CDW and HAA generation are shown over the fermentation time. The black filled rectangles and the dashed line depict HAA titers, while the gray triangles represent the biomass concentrations. The error bars represent deviation from the mean of two replicates

After transforming *P. putida* KT2440 with plasmid pPS05, the bacterium produced a mixture of different mono-rhamnolipid congeners, which were identified by HPLC–MS/MS analysis. 63% of the total mono-rhamnolipids consisted of the Rha-C_10_-C_10_, a rhamnolipid with one rhamnose and two β-hydroxy-fatty acids with ten carbon atoms each. A further 19% were composed of the Rha-C_10_-C_12_, while the third biggest fraction (16%) was the Rha-C_10_-C_12:1_. The smallest fraction with only 2% was the Rha-C_8_-C_10_ (Fig. 2a). Interestingly, as found by Behrens et al. [41] the shorter β-hydroxy-fatty acid chain is always in the first position, being attached to the rhamnose molecule. The total HAA share was around 1% of total biosurfactant content (not shown).

The *P. putida*-based cell factory produced a titer of 2.4 g/L rhamnolipids in LB medium supplemented with 11 g/L glucose. The carbon yield was about 35% [Cmol_RL_/Cmol_Glc_], which is approximately 49% of the maximal theoretical yield.

#### Di-rhamnolipid production

After transforming *P.* *putida* KT2440 with the *rhlC* containing vector, the new microbial cell factory was able to produce di-rhamnolipids.

The total biosurfactant titer was 3.3 g/L, corresponding to a carbon yield of 49% [Cmol_RL_/Cmol_Glc_]. The experiment was carried out in complex LB medium supplemented with 10 g/L glucose, representing 68% of the maximal theoretical yield (Table 1).

**Table 1:** Fermentation characteristics of the three engineered recombinant biosurfactant producers (*CDW* cell dry weigth, *SF* surfactant, *Glc* glucose)

Organism	Glucose [g/L]	Cell dry weight [g_CDW_/L]	Maximal titer [g_SF_/L]	Yield [g_SF_/ g_Glc_]	Carbon yield^a^ [Cmol_SF_/ Cmol_Glc_]	Production time^b^ [h]	Specific surfactant-production rate^c^ [g_SF_/(g_CDW_ h)]
*P. putida* KT2440 pPS05	11	3.4	2.40	0.23	0.35 (49%)	23	0.031
*P. putida* KT2440 pWJ02	10	1.8	3.26	0.33	0.49 (68%)	48	0.038
*P. putida* KT2440 pSB01	10	5.7	1.54	0.15	0.27 (41%)	22	0.012

^a^For the calculation of yields during production on complex media, rhamnolipids and HAAs were assumed to be synthesized from the used carbon source, while media compounds were utilized for cell growth. The numbers in parenthesis show the percentage of the maximal possible theoretical yield reached

^b^The production time is the time past until the maximal titer was reached

^c^The specific production rates were calculated as average over the fermentation time until the peak point was reached

The rhamnolipids produced by this cell factory are a mixture as in the production with the native producer. Of the total biosurfactants, 86% were di-rhamnolipids, 13% were mono-rhamnolipids, while only about 1% were HAAs. The spectrum of side chain lengths in synthesized congeners did not differ significantly from the observed distribution in mono-rhamnolipid production (Fig. 2a). The fraction of the main congener (C_10_-C_10_) was 65%, while C_10_-C_12:1_ and C_10_-C_12_ amounted to 15–19% and the minor C_10_-C_8_ only 1%. In total, 86% of the incorporated β-hydroxy-fatty acids possessed 10 carbon atoms, while only 13% featured 12 carbon atoms. C_8_ β-hydroxy-fatty acids occurred to only less than 1%.

#### HAA production

A third biosurfactant producing cell factory was constructed by only using the first enzyme from rhamnolipid synthesis.

*Pseudomonas putida* KT2440 was transformed with pSB01, the plasmid to produce HAAs. After 22 h of growth, an HAA titer of 1.5 g/L was reached with a cell density of around 6 g/L. This results in a carbon yield of 27% [Cmol_HAA_/Cmol_Glc_], which is 40% of the theoretical yield. In contrast to rhamnolipids, HAAs are taken up by the cell after the carbon source (here glucose) is depleted (Fig. 2b), which resulted in zero free HAAs 25 h after peak production.

The distribution of the four congeners was similar to the ratios described for mono- and di-rhamnolipids. The C_10_-C_8_ content was slightly elevated (5%), but the other three ratios remained in the previously determined ranges (Fig. 2a).

Compared with the two rhamnolipid microbial cell factories it becomes obvious that there is room for improvement of recombinant HAA production (Table 1). Rhamnolipid titers ranged around 3 g/L, while the HAA concentration was only half of that, despite the fact that synthesis of the precursor rhamnose is not required. One reason might be that *P.* *putida* is capable of degrading HAA. As soon as the external carbon source is consumed, HAAs are taken up to supply a carbon source for metabolism as was also shown by Wittgens et al. [40] (Fig. 2b).

Another important difference is the production rate, which was highest in the case of mono-rhamnolipid production (0.047 g_RL_/(g_CDW_ h)). While almost the same amounts of rhamnolipids were produced, reaching this titer took twice as long for di-rhamnolipid production. The specific di-rhamnolipid production rate was only one fourth lower, because biomass formation was lower in the microbial cell factory *P. putida* pWJ02.

The carbon yield was high in all three cell factories. In HAA production 27% [Cmol_HAA_/Cmol_Glc_] was reached compared to 40 and 49% [Cmol_RL_/Cmol_Glc_] in mono- and di-rhamnolipid production, respectively. These values translate to 40% and up to 70% of the maximal achievable yield. These yield values are high for cell factories without improvements of metabolic operation, reinforcing the proposed “driven by demand” principle [39], which proposes that high specific yields can result simply by creating artificial demand via strong expression of enzymes at metabolic endpoints.

#### Biosurfactant mixture

The cell factories described above produce rhamnolipids differing in the number of rhamnose moieties, but the mixture of hydroxy-fatty acid chain lengths remains constant. Next, the adjustment of the ratio of the specific biosurfactants in the mixture was approached. When comparing the di-rhamnolipid producing microbial cell factory to a previously constructed di-rhamnolipid synthesis plasmid [40] a striking discrepancy becomes evident. With all other conditions the same, the stronger expression system in *P.* *putida* KT2440 pWJ02 resulted in about an 80% higher rhamnolipid titer than *P.* *putida* KT2440 pVLT33_*rhlABC* (1.6 g/L instead of 0.9 g/L) at slightly lower end cell dry weight (CDW) concentrations. The share of the precursor HAA was consistent, but the di-rhamnolipid content increased in the *P.* *putida* KT2440 pWJ02 to 77% (from 66% in *P.* *putida* KT2440 pVLT33_*rhlABC*). The congener distribution (based on chain lengths) remained unchanged. Importantly, the 80% increased rhamnolipid concentration is completely due to more synthesized di-rhamnolipids, since the mono-rhamnolipid concentration was the same in both cultivations. Higher di-rhamnolipid production can most likely be attributed to a strongly increased *rhlC* transcriptional activity due to the insertion of an additional promoter upstream of *rhlC*. It is thus possible to actively adjust the share of di-rhamnolipids in the biosurfactant mixture, by fine-tuning the transcriptional strength via used promoters.

#### Pulsed fed-batch cultivation

To intensify the cultivation, a fed-batch approach was carried out. Instead of fermenters with the known challenges of excessive foaming [45, 46] Fernbach flasks were used. The respective production organism (*P.* *taiwanensis* VLB120 pSB01 for HAA production, *P. putida* KT2440 pPS05 for mono-rhamnolipid production and *P. putida* KT2440 pWJ02 for di-rhamnolipid production) was cultivated in LB medium supplemented with 10 g/L glucose (the data for HAA production is shown here exemplarily). In contrast to rhamnolipids, HAAs are degraded by *Pseudomonas* when the carbon source is limiting, causing a challenge for an optimal time of harvest. To avoid carbon limitation and HAA degradation, the glucose concentration was aimed to be kept at 1 g/L and higher.

The experiment was performed for almost a week in which 10 mL of 50% (w/v) glucose solution were fed four times. HAA titers increased steadily during the whole experiment, while the optical density increased slowly after the exponential growth phase. This increase in optical density could reflect polyhydroxyalkanoate formation and not cell growth [47]. The glucose uptake rate consistently decreased after the cells ceased to grow, indicating that non-growing cells are able to sustain HAA production. Notably, an HAA titer of 7 g/L was reached.

#### Biosurfactant production from alternative carbon sources

**Fig. 3:**
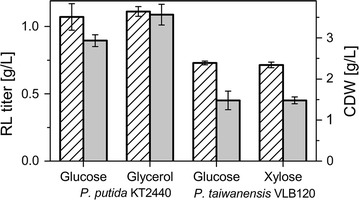
Mono-rhamnolipid production using alternative carbon sources. CDW and rhamnolipid titers are presented in g/L (striped columns and grey columns, respectively). The error bars represent the deviation from the mean of two biological replicates

Glycerol and xylose were chosen as alternatives for glucose, but *P.* *putida* cannot use xylose as carbon source. Instead *P. taiwanensis* VLB120, which uses the Weimberg pathway [48], was transformed with pPS05 enabling it to produce rhamnolipids directly from xylose. Rhamnolipid production was possible on both glycerol and xylose. Compared to glucose, titers and end CDWs were not significantly decreased (Fig. 3).

### Biosurfactant purification

To determine whether the different biosurfactant mixtures actually have variable physico-chemical properties, they had to be recovered in high purity from the cultivation broth. Therefore, a new procedure based on filtration and adsorption/desorption was developed.

At pH 7 the final product had a highly viscous appearance. Increasing the pH of the supernatant to 10 prior to the adsorption resulted in an improved texture of the product, when it could be dried to a fine powder. At higher pH the biosurfactants were no longer present as acids but as salts, which might have caused the altered appearance.

In the following paragraphs the purification of the HAAs, the mono-, and the di-rhamnolipids using the adsorption/desorption procedure is described.

#### HAA purification

**Fig. 4:**
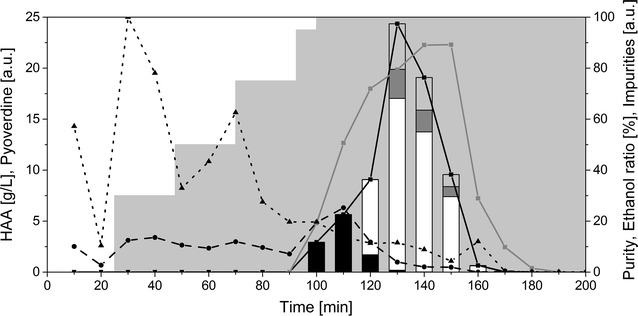
Purification of HAAs using an adsorption/desorption procedure. The composition of the different fractions collected is shown. Each fraction corresponds to 10 min of elution. The black curve with the squares displays the total HAA concentration in g/L. The black dashed line with the circles shows the pyoverdine content in arbitrary units, while the black dotted line with triangles represents other impurities measured by absorption at 400 nm in arbitrary units. The gray line with squares displays the purity. The grey area in the background shows the composition of the elution solution with stepwise increasing ethanol concentrations. The stacked bars display the congener’s composition of the HAAs. Black bars: C_10_-C_8_, white bars: C_10_-C_10_, dark grey bars: C_10_-C_12:1_, light grey bars: C_10_-C_12_ (all in g/L)

The first C_10_-C_8_ HAA congener eluted at 75% ethanol, resulting in two fractions only containing this congener (100 and 110 min) (Fig. 4). The purity of these fractions was low at about 20 and 50%, possibly because of pyoverdines eluting at the same time. In the third biosurfactant-containing fraction, two congeners were included (C_10_-C_8_ and C_10_-C_10_) followed by three congeners eluting simultaneously. The highest purity of these fractions was around 90% (140 and 150 min). As noticed above, the pyoverdines co-eluted mostly with the C_10_-C_8_ congener. The first fractions (until 80 min) contained most of the colored substances. Separation of these mostly unspecified compounds from the biosurfactant-containing fractions was thus successful.

#### Mono-rhamnolipid purification

**Fig. 5:**
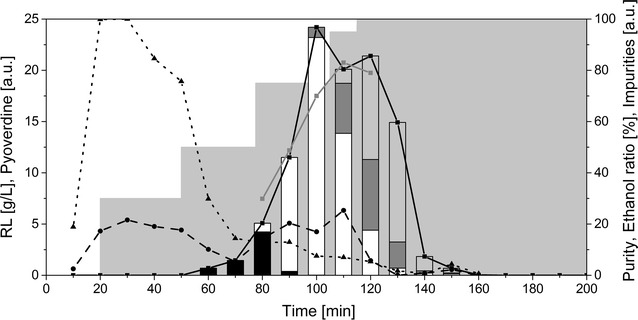
Purification of mono-rhamnolipids using an adsorption/desorption procedure. The composition of the different fractions collected is shown. Each fraction corresponds to 10 min of elution. The black curve with the squares displays the total rhamnolipid concentration in g/L. The black dashed line with the circles shows the pyoverdine content in arbitrary units, while the black dotted line with triangles represents other impurities measured by absorption at 400 nm in arbitrary units. The gray line with squares displays the purity. The grey area in the background shows the composition of the elution solution with stepwise increasing ethanol concentrations. The stacked bars display the congener’s composition of the rhamnolipids. Black bars: Rha-C_8_-C_10_, white bars: Rha-C_10_-C_10_, dark grey bars: Rha-C_10_-C_12:1_, light grey bars: Rha-C_10_-C_12_ (all in g/L)

Compared to the elution profile of the HAAs, the mono-rhamnolipids eluted at a lower ethanol concentration (Fig. 5) due to their higher hydrophilicity caused by the additional sugar group. The purification resulted in an even better separation of the congeners when compared with HAAs. Again, the Rha-C_8_-C_10_ congener eluted prior to all other congeners (60, 70, and 80 min). Afterwards the major congener (Rha-C_10_-C_10_) eluted (90 min). Moreover, a fraction containing mostly Rha-C_10_-C_12_ congeners (130 min) could be collected. The highest purity was about 83% (110 min). The elution of the pyoverdines followed a pattern similar to the HAA purification experiment, eluting in fractions two to six and again from 80 to 120 min. The color measurement delivered also similar results to the above results (Additional file 1: Figure S2). It can thus be concluded that in the case of mono-rhamnolipids the adsorption/desorption delivers a good separation of the biosurfactants from impurities.

#### Di-rhamnolipid purification

**Fig. 6:**
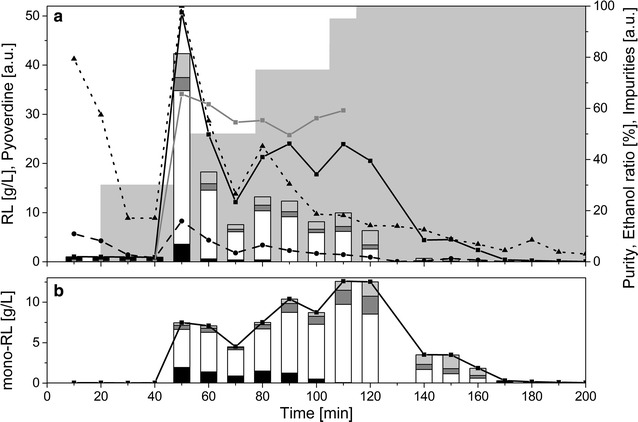
Purification of di-rhamnolipids using an adsorption/desorption procedure. **a** The composition of the different fractions collected is shown. Each fraction corresponds to 10 min of elution. The black curve with the squares displays the total rhamnolipid (mono and di) concentration in g/L. The black dashed line with the circles shows the pyoverdine content in arbitrary units, while the black dotted line with triangles represents other impurities measured by absorption at 400 nm in arbitrary units. The gray line with squares displays the purity. The grey area in the background shows the composition of the elution solution with stepwise increasing ethanol concentrations. The stacked bars display the congener’s composition of the di-rhamnolipids. Black bars: Rha-Rha-C_8_-C_10_, white bars: Rha-Rha-C_10_-C_10_, dark grey bars: Rha-Rha-C_10_-C_12:1_, light grey bars: Rha-Rha-C_10_-C_12_ (all in g/L). **b** Mono-rhamnolipids contained in the fractions. The black line depicts the total mono-rhamnolipid concentration, while the stacked bars represent the amount of mono-rhamnolipid congeners. Black bars: Rha-C_8_-C_10_, white bars: Rha-C_10_-C_10_, dark grey bars: Rha-C_10_-C_12:1_, light grey bars: Rha-C_10_-C_12_ (all in g/L)

The di-rhamnolipids eluted at even lower ethanol concentrations because of their even higher hydrophilicity (Fig. 6a). Unfortunately, separation of congeners was not as successful as observed before. No fraction containing only the Rha-Rha-C_8_-C_10_ could be captured and the later fractions contained all of the remaining three congeners. Furthermore, the pyoverdines and the impurities measured by the color were not separated well from the biosurfactant-containing fractions. Remarkably, the rhamnolipid fractions spread out over more than 70 min, which was unique to the di-rhamnolipid purification. It can also be seen that the mono-rhamnolipids were not separated from the di-rhamnolipids (Fig. 6b). The purities of the prepared di-rhamnolipids reached about 60%, which was significantly lower than the values achieved for the HAAs and the mono-rhamnolipids. In total, the desorption procedure for the di-rhamnolipids has to be substantially enhanced to achieve a satisfying purification. The choice of an optimal adsorbent has a decisive effect on the effectivity of this purification procedure as was shown earlier [49].

### Biosurfactant properties

Required biosurfactant properties depend on the specific application. To tailor the biosurfactant, the physico-chemical properties have to be known. Here, the divergent properties of nearly pure HAAs, mono-, and di-rhamnolipids (90, 80, and 65% purity, respectively) were characterized.

#### Foam formation

**Fig. 7:**
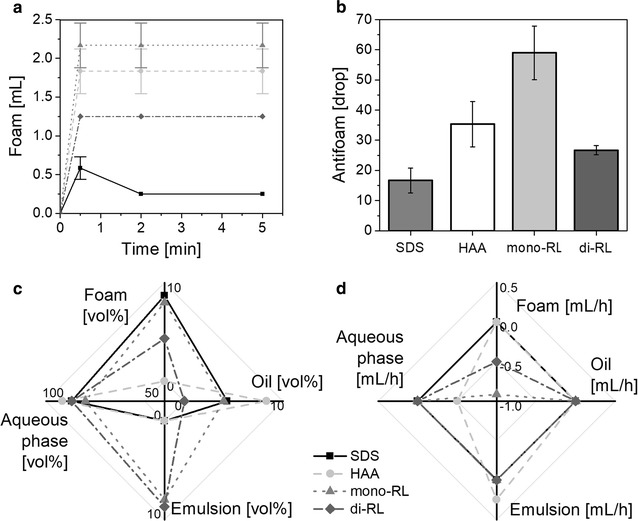
Biosurfactant properties. **a** Foaming capabilities I. Course of the foam formation over time. HAAs are represented by the light grey dashed line with circles, mono-rhamnolipids by the dotted grey line with triangles, di-rhamnolipids by the dark grey line with diamonds, SDS is depicted by black line with squares and serves as a reference. **b** Foaming capabilities II. Amount of added antifoam during the testing of the antifoam effectiveness. The error bars represent the deviation of the mean of three experiments. **c** Emulsification capability. Volumetric percentage of the different phases foam, oil, emulsion, and aqueous phase of the initial emulsion. **d** Emulsion stabilization. Volumetric change over time of the different phases foam, oil, emulsion, and aqueous phase

The results of the foam formation and the antifoam effectiveness tests indicated that both the foam formation and the foam stabilization were strongest for mono-rhamnolipids (2 mL foam volume; 59 drops of antifoam) followed by HAAs (1.8 mL foam volume; 35 drops of antifoam) and di-rhamnolipids (1.2 mL foam volume; 27 drops of antifoam) (Fig. 7). All three biosurfactants displayed significantly stronger foam formation than the reference SDS, which is a strong foaming agent. Specifically, SDS only formed 0.5 mL foam, which then also collapsed over the time of testing.

#### Emulsion stability

The initial emulsion formed with oil and water using the three biosurfactants demonstrated that SDS and HAA were less capable of forming an emulsion than mono- and di-rhamnolipids (Fig. 7c). Specifically, the resulting emulsions with SDS and HAA were mainly split into an oil (SDS: 4.4 vol%; HAA: 8.3 vol%) and an aqueous phase (SDS: 87 vol%; HAA: 92 vol%), while the emulsion phase was absent. On the other hand, the volumetric percentage of the emulsion phase for mono- and di-rhamnolipids amounted to 8 vol%. Especially di-rhamnolipids showed high emulsion properties: Whereas much of the oil phase was in foam for mono-rhamnolipids, it was completely emulsified with di-rhamnolipids (Fig. 7c).

Regarding the stability of emulsions formed with oil and water using the three biosurfactants, the emulsion with the HAA-surfactant was clearly less stable than the emulsion with mono- or di-rhamnolipids (Fig. 7d). While only a change in foam volume for the emulsion with mono- and di-rhamnolipids was observed (mono-rhamnolipids: − 0.92 mL/h, di-rhamnolipids: − 0.50 mL/h), for the emulsion with HAA a change in volume of the aqueous and emulsion phase over time was detected (aqueous phase: − 0.50 mL/h; emulsion phase: 0.25 mL/h). The emulsion stability of the solution with di-rhamnolipids was highest since the volumetric change of the foam was lower than that of the solution with mono-rhamnolipids (Fig. 7d).

The emulsion stability result is strongly correlated with the outcome of the coagulation test. Here, the lowest coagulation was observed for mono-rhamnolipids followed by di-rhamnolipids, while the strongest coagulation was observed for SDS and HAAs.

These results match with previous studies, where rhamnolipids were found to feature excellent emulsification activities [50, 51].

#### Critical micelle concentration

The critical micelle concentration (CMC) based on surface tension was determined for all three biosurfactants. The lowest final surface tension was for HAAs with 25.4 mN/m, followed by mono-rhamnolipids (27.5 mN/m), and di-rhamnolipids (31.8 mN/m) (the detailed courses of the surface tension can be seen in Additional file 1: Figure S3). The CMCs are 113 mg/L for the HAAs, 124 mg/L for the mono-rhamnolipids, and 148 mg/L for the di-rhamnolipids. Notably, no surface tension could be measured with HAAs at concentrations higher than 500 mg/L as the spotted drops on the plate collapsed due to very low surface tension. Hence, the slope of the regression line for the concentrations beneath CMC is expected to be steeper, which would translate to a lower CMC for HAAs as here reported.

The property determination specific for the three surface-active compounds reveals that indeed the investigated biosurfactants differ in their properties and can thus potentially be used for different applications. If, for example, an application requires foaming, e.g., in shampoos it will be beneficial to use mono-rhamnolipids. If, emulsification is of interest, di-rhamnolipids would be the better choice. It has to be noted that the di-rhamnolipids are actually a mixture including mono-rhamnolipids. Pure di-rhamnolipids would thus probably deliver even better emulsification properties. The HAAs clearly are best suited if the aim is to lower the surface tension in a specific application. Thus, a first approach for designing biosurfactants for specific applications was demonstrated.

However one has to keep in mind the purity of the applied biosurfactants. While for mono-rhamnolipids and HAAs the purity was above 80%, for di-rhamnolipids it was significantly lower with 65%. These differences might also play a role in the determination of the specific properties. When purer samples can be obtained these experiments should be repeated.

## Discussion

### Designer biosurfactants

To synthesize designer biosurfactants, we here pursued a strategy based on genetic engineering of *Pseudomonas* cell factories to yield specific biosurfactant molecules. However, instead of producing novel, new-to-nature biosurfactants, we merely exploit the structural diversity already existing in nature [19]. Rhamnolipids are always produced in mixtures where both the chain length of the β-hydroxy-fatty acids and the number of attached rhamnose molecules differs. Typically, rhamnolipid producing bacteria produce up to eight congeners (and more in traces). In this study, we reduced this diversity by only using two rhamnosyltransferases.

The basic concept of designer rhamnolipid production was previously reported [52]. The authors sketched two different strategies for mono-rhamnolipid synthesis. The first was the recombinant expression of *rhlAB* in a suitable host organism, which has been successfully carried out by Ochsner et al. [53] but only very low mono-rhamnolipid titers could be reached using *E. coli*. To our knowledge, this is the first work to achieve production of high titers of mono-rhamnolipids, a rhamnolipid mixture of *P.* *aeruginosa* type (mono- and di-rhamnolipids), and HAAs in one recombinant production host.

### Chain length specificity

**Fig. 8:**
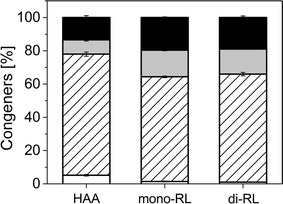
Congener composition of the biosurfactant mixtures produced by the three different production cell factories. The first bar represents the composition in the HAA producing cell factory carrying only *rhlA*. The second cell factory also carries *rhlB* and is thus capable of producing mono-rhamnolipids, while the third cell factory produces di- and mono-rhamnolipids, mediated by the second rhamnosyltransferase *rhlC*. White columns depict the share of the congener being composed of C_10_-C_8_ β-hydroxy-fatty acids. The striped box represents the share of C_10_-C_10_ hydrophobic moieties, while the grey and black fields stand for C_10_-C_12:1_ and C_10_-C_12_ hydrophobic moieties of the biosurfactant molecule, respectively. The error bars represent the deviation from the mean and are based on the values of ten time points from two biological replicates

While the assembly of the hydrophilic moiety of the rhamnolipid is well understood and adjustable by genetic engineering, the mechanism controlling the length of the β-hydroxy-fatty acids of the biosurfactant remains to be discovered. Nevertheless, this work provides some insight on this topic. For example, the overall trend in the fractions of the different congeners in the total biosurfactant mixture (Fig. 8) seems to be consistent throughout the three production backgrounds: The main congener contains two C_10_ β-hydroxy-fatty acids, while the two congeners having C_12_ chains roughly have similarly shares. The smallest fraction is made up of the congener containing a C_8_ β-hydroxy-fatty acid. These results suggest that the specificity for the chain length of the assembled β-hydroxy-fatty acids mainly lies with the RhlA enzyme, with a strong preference for C_10_. RhlB seems to discriminate against C_8_ HAAs, as the congener distribution between HAAs and mono-rhamnolipids vary slightly (Fig. 8). RhlC has no influence on rhamnolipid congener distribution.

Nevertheless, the decisive step in chain length specificity is most likely the condensation of the two β-hydroxy-fatty acids. On glucose as carbon source, we hypothesize that the RhlA enzyme detracts the activated β-hydroxy-fatty acids from fatty acid de novo synthesis and has a high affinity for C_10_ carbon chains, with minor activity for C_8_, C_12_, and C_12:1_ β-hydroxy-fatty acids. This hypothesis is also supported by comprehensive studies investigating the RhlA enzyme in vitro as well as in vivo [40, 54], while only few authors found a specificity in the RhlB enzyme [55]. Our findings further contradict any involvement of the rhamnosyltransferases in chain length specificity. If RhlB specifically had a lower affinity to C_10_-C_8_ HAAs these molecules would be accumulating. However, this is not reflected in the experimental data (not shown). We thus conclude that chain length preference is almost exclusively ascribed to RhlA.

### Structure property relation

From the experiments determining the properties of the different surfactant molecules we conclude that mono-rhamnolipids form the most stable emulsions, which can be explained by the amphiphilicity of the molecules of interest. While di-rhamnolipids have a highly hydrophilic moiety (two rhamnose residues), the hydrophilicity of HAAs is only due to the ester, the carboxyl, and the hydroxy groups. Thus, the accumulation of di-rhamnolipids at surfaces with different hydrophobicities will be higher compared to the less amphiphilic molecule HAA, thereby stabilizing emulsions more effectively. Consistently, the effectivity of mono-rhamnolipids for emulsification lies between the other two molecules because of their intermediate amphiphilicity resulting from one rhamnose residue.

The findings of different foaming capabilities are somewhat counterintuitive, as the higher amphiphilicity of the di-rhamnolipid molecule should translate into higher surface activity and hence foaming. The discrepancy can most likely be explained by the lower purity of the di-rhamnolipid sample.

The determined CMCs (113 mg/L for the HAAs, 124 mg/L for the mono-rhamnolipids, and 148 mg/L for the di-rhamnolipids) range between previously observed CMCs (5 mg/L [56] to 230 mg/L [57]). Most reported values however are lower than the CMCs calculated here [11]. A possible reason might be differences in the purities of the used samples.

As suggested for the other measurements, the differences in the CMCs can be explained by the different hydrophilicity caused by the molecular structures. With two rhamnose residues, di-rhamnolipids are the most hydrophilic components, while HAAs without sugar residues are the least hydrophilic. The lower the hydrophilicity, the faster the molecules form micelles. In addition, the HAAs accumulate preferably at the surface (or interface, if present), rather than in the water phase. As they are smaller, more molecules can accumulate at the same surface, resulting in a lower final surface tension compared to the other molecules.

## Conclusions

For the first time, we demonstrated microbial synthesis of designer biosurfactants by metabolic engineering of a *Pseudomonas* cell factory. While properties of biosurfactants (synthetic and biological) can easily be adjusted by chemical modification this has not been shown by means of genetic engineering of the organism so far. We were not only able to produce three different biosurfactant mixtures differing in the number of the attached rhamnose molecules, but could also adjust the share of the mono-rhamnolipids in the di-rhamnolipid mixture.

We furthermore established an easy purification procedure that was efficient for all produced biosurfactants and could show that the properties of the produced mixtures indeed significantly deviate from one another.

Finally, we demonstrated the production and purification of the precursor for rhamnolipid synthesis, the HAA. Our results indicate that this molecule can also be used in specific biosurfactant applications.

This study is therefore a first step for the production of designer biosurfactants tailored for specific applications.

## Additional file

**Additional file 1.** Compilation of extra information for better comprehension of the contents of the study. The file contains a more detailed description of some methods, as well as some additional figures for the presented results.

## Abbreviations

CDW: cell dry weight
RL: rhamnolipid
HAA: hydroxyalkanoyloxy alkanoate
LB: Lysogeny Broth
