# Supplementary material for: Designer rhamnolipids by reduction of congener diversity: production and characterization
Source: Microb Cell Fact. 2017 Dec 14;16:225. doi: 10.1186/s12934-017-0838-y (PMC5729600; doi:10.1186/s12934-017-0838-y)
Supplement: Supplementary file 1 — Additional file 1. Compilation of extra information for better comprehension of the contents of the study. The file contains a more detailed description of some methods, as well as some additional figures for the presented results. [file 12934_2017_838_MOESM1_ESM.docx]

# Materials and Methods

## Bacterial strains, culture conditions, and plasmids

Table S-1. Bacterial strains used for biosurfactant production in this work.

| **Strain** | **Plasmid** | **Genes** | **Fermentation products** | **Reference** |
| --- | --- | --- | --- | --- |
| *P. putida* KT2440 | - |  | - | [1] |
| *P. taiwanensis* VLB120 | - |  | - | [2] |
| *E. coli* DH5α | - |  | - | [3] |
| *P. putida* KT2440 | pPS05 | *rhlA*, *rhlB* | mono-rhamnolipids | [4] |
| *P. putida* KT2440 | pWJ02 | *rhlA*, *rhlB*, *rhlC* | di-rhamnolipids, mono-rhamnolipids | This work |
| *P. putida* KT2440 | pVLT31_rhlABC | *rhlA*, *rhlB*, *rhlC* | di-rhamnolipids, mono-rhamnolipids | [5] |
| *P. putida* KT2440 | pSB01 | *rhlA* | HAAs | This work |
| *P. taiwanensis* VLB120 | pSB01 | *rhlA* | HAAs | This work |
| *P. taiwanensis* VLB120 | pPS05 | *rhlA*, *rhlB* | mono-rhamnolipids | This work |

## Construction of expression vectors

Table S-2. Plasmids used within this work.

| **Name** | **Backbone** | **ori** | **Resistance** | **Genes** | **Reference** |
| --- | --- | --- | --- | --- | --- |
| pBBR1 | pBBR1 | BBR1 | Tetracycline | - | [6] |
| pSEVA241 | pSEVA241 | RO1600/ColE1 | Kanamycin | - | [7] |
| pPS05 | pBBR1 | BBR1 | Tetracycline | *rhlA*, *rhlB* | [4] |
| pVLT31_*rhlC* | pVLT31 |  | Tetracycline | *rhlC* | [5] |
| pVLT31_*rhlABC* | pVLT31 |  | Tetracycline | *rhlA*, *rhlB*, *rhlC* | [5] |
| pWJ02 | pBBR1 | BBR1 | Tetracycline | *rhlA*, *rhlB*, *rhlC* | This work |
| pSB01 | pSEVA241 | RO1600/ColE1 | Kanamycin | *rhlA* | This work |

Table S-3. PCR primers used in this work. The RBS is printed in bold, restriction sites are underlined.

| **Name** | **Direction** | **Used for** | **Template** | **Contains** | **Sequence** |
| --- | --- | --- | --- | --- | --- |
| PS13 | fwd | pWJ02 | pVLT31_*rhlC* | AscI, SphI, SynPro8, RBS | GCGGCGCGCCAATATTGCATGCGTTGATTGACAA  AGCGCTTACCTCTTTCTATAATATAGAGTGTACA  **AGGGGG**GAGCTCATGGACCGGATAGACATGGG |
| PS14 | rev | pWJ02 | pVLT31_*rhlC* | AscI, HindIII | GCGGCGCGCCAAGCTTCTAGGCCTTGGCCTTG |
| SB01 | fwd | pSB01 | pPS04 | KpnI | ATCCAGGGTACCAGCTCTTG |
| SB02 | rev | pSB01 | pPS04 | SphI | CTGCATGCCTAGGCTTGTCAAGGAAAGG |

## Biosurfactant quantification

Cells and cell residues were separated by centrifugation at 13,000 x g for 2 min. 500 µL of the supernatant were then mixed with 500 µL of acetonitrile and vortexed. Subsequently, samples were incubated overnight at 4°C to facilitate precipitation of any residual material that might clog the column. The next day, the samples were centrifuged again and the supernatant was filtered with a Phenex-RC (regenerated cellulose) syringe filter (diameter 4 mm, pore size 0.2 µm) (Phenomenex, Aschaffenburg, Germany).

Samples were analyzed on an UltiMate3000 series HPLC. The system is composed of the pump LPG-3400SD, the autosampler WPS-3000 (RS), and the column oven TCC-3000 (RS), with a Corona CAD detector (all Thermo Fisher Scientific Inc., Waltham, MA, USA). The CAD was supplied with a continuous nitrogen stream by the nitrogen generator Parker Balston NitroVap-1LV (Parker Hannifin GmbH, Kaarst, Germany).

For separation of rhamnolipid species and derivatives, a NUCLEODUR C18 Gravity column from Macherey-Nagel GmbH & Co. KG (Düren, Germany) with a particle size of 3 µm in the dimensions of 150 x 4.6 mm was used. This column was protected by a guard column (4 x 4.6 mm, particle size 3 µm). The gradient started with 70% of acetonitrile and 30% purified water containing 0.2% formic acid. After 1 minute, acetonitrile was increased to 100% within 8 min. After 11 min total analysis time, acetonitrile was decreased to 70% during 1 min. The total analysis time was 15 min. The column oven temperature was set to 40°C, and the injection volume to 5 µL. The flow rate was 1 mL/min.

## Biosurfactant identification

For identification of rhamnolipid congeners additional to the LC-MS/MS measurements we used gas chromatography coupled to tandem mass spectrometry (GC-MS/MS) to analyze the fatty acid moiety of the rhamnolipid molecule. To this end, the rhamnolipid first had to be hydrolyzed to cleave of the rhamnose residue. Subsequently fatty acids were converted to the respective fatty acid methyl esters (FAME) for GC analysis. This procedure is partly based on the method developed by Monteiro *et al*. [8].

The hydrolysis was carried out by adding 1 mL of 6 M HCl to about 5 mg purified sample. The mixture was hydrolyzed at 120°C for 1.5 h in a 2 ml sealed ampoule bottle.

The fatty acids were then extracted with 3 mL diethyl ether. The extract was dried and the residue was esterified in 1 mL 10 % H_2_SO_4_-methanol solution at 55°C overnight. Subsequently, 5 mL of purified water were added to the reaction mixture, followed by extracting of the FAME. This was done by extracting three times with 3 mL diethyl ether. The diethyl ether was evaporated at room temperature. Subsequently the FAME were dissolved in 1 mL methanol before the sample was analyzed by GC-MS/MS. We used the TRACE GC coupled to the TSQ Triple Quadrupole MS (both Thermo Fisher Scientific Inc., Waltham, MA, USA) with the HP-5MS capillary column (30 m × 0.25 mm × 0.25 µm). The course of the oven temperature was as follows: Initially is was held at 80°C for 2 min and then increased to 280°C at a rate of 10°C/min and kept for 12 min. 1 µL sample were injected using splitless mode. The carrier gas was helium at a flow of 0.9 mL/min.

## Determination of biosurfactant properties

### Foam formation

To determine the ability to generate foam, 5 mL of a 0.5 g/L biosurfactant solution was filled into a 15 mL falcon tube and shaken for 2 min at 300 rpm and 30°C with a shaking diameter of 50 mm on a Multitron shaker by Infors AG (Bottmingen, Switzerland). The volume of the foam was read out after 30 s, 2 min, and 5 min.

### Emulsion stability

This test determines coagulates formed in an oil/water emulsion, with higher values indicating that the resulting emulsion is not as stable. To determine stability of emulsions, 5 mL of a 0.5 g/L concentrated biosurfactant solution was filled into a 15 mL falcon tube and 500 µL rapeseed oil was carefully added. The initial emulsion was determined by inverting the tube once and measuring the volumes of oil, foam, emulsion, and aqueous phase after 30 s. Subsequently the tube was inverted ten times and stored at 30°C to define the emulsion stability after 30 min, 2 h, and 24 h by measuring the respective volumes. To allow a comparison between the results, we calculated the volumetric change of the different phases over time for each biosurfactant. Therefore, the volumes of the respective phases for each biosurfactant were plotted over the time and the linear regression function was determined subsequently.

### Antifoam effectiveness

For this test a special construction consisting of 50 mL falcon tubes, a 50 mL syringe, and a filter with a pore size of 25 mm and a polycarbonate membrane from Nucleopore GmbH (Tübingen, Germany) was applied (Figure S-1). With this device, a flow of pressurized air could be applied to the biosurfactant solution in a steady manner while observing foam formation. This device was mounted on a tripod and connected to an air supply. An air flow of 186 L/h was maintained with a pressure reducer. 30 mL of a 0.5 g/L concentrated biosurfactant solution were filled into the falcon tube-syringe construction. Whenever the foam reached the 60 mL mark, one drop of antifoam 205 by Sigma-Aldrich GmbH (St. Louis, Missouri, USA) was added with a Pasteur pipette. This procedure was carried out for 30 min and the number of added antifoam drops was counted.


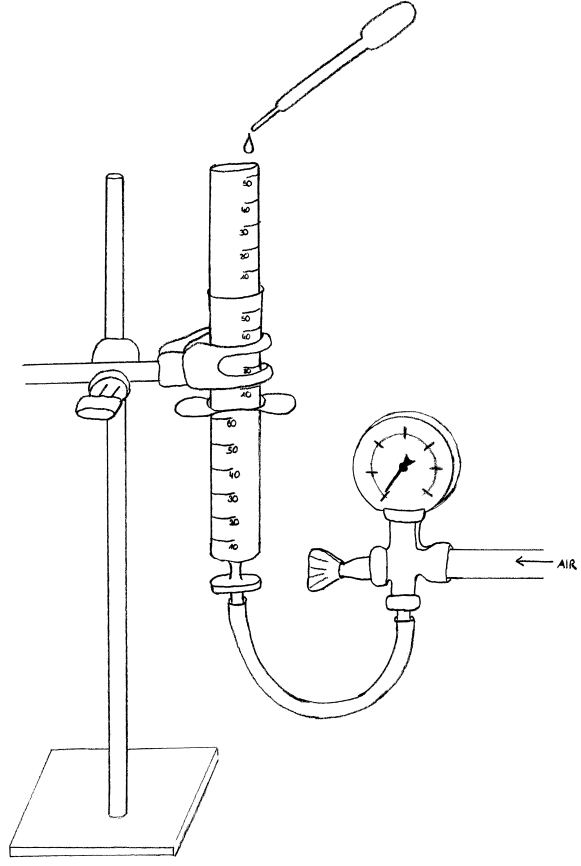


Figure S-1. Experimental setup for the determination of antifoam effectiveness. The construction was mounted on a tripod and consisted of 50 mL falcon tubes, a 50 mL syringe, and a filter, which was connected to an air supply via a pressure reducer.

### Coagulation

To measure coagulation, 3 mL of rapeseed oil was added to 30 mL of a 0.5 g/L biosurfactant solution in a 100 mL Schott flask. This solution was stirred with 300 rpm for 30 min in a 60°C water bath. The emulsion was filtered with a cellulose nitrate filter with a pore size of 0.45 µm by Sartorius Stedim Biotech GmbH (Göttingen, Germany) and a vacuum pump. After drying the filter at 60°C for three days, it was weighed.

### Critical micelle concentration

To determine the critical micelle concentration (CMC), different concentrations of the three purified biosurfactants (HAA, mono-rhamnolipid, and di-rhamnolipid) were adjusted by diluting with bi-distilled water. The surface tension of the water was checked to ensure there were no surface-active effects. All dilutions were prepared in triplicates.

The surface tension was measured using the Wilhelmy plate method (Krüss K11 force tensiometer, Krüss GmbH, Hamburg, Germany) and a small curved plate (Krüss PL02). 600 µL of the rhamnolipid solution were spotted on a metal plate and the measurement was started. Between two measurements, the plate was cleaned by rinsing it with bi-distilled water prior to rinsing it with ethanol. The ethanol was wiped away and potential remains were allowed to evaporate. Afterwards, the small curved plate was cleaned similarly. After rinsing with ethanol, the plate was heated using a Bunsen burner until it glowed.

The CMC was determined by plotting the measured surface tensions against the logarithmic biosurfactant concentration. Two regions with different slopes were identified visually and fitted with two individual curves. The CMC was calculated at the intersection of the two extrapolated lines.

# Results

### Mono-rhamnolipid purification

Figure S-2. Colors of the fractions from the mono-rhamnolipid purification from minute 10 to 150.

### Critical micelle concentration

Figure S-3. Measured surface tensions and corresponding CMC values for different biosurfactants. A) – C) Surface tensions of the biosurfactants HAA (A), mono-rhamnolipid (B), di-rhamnolipid (C), respectively, at different concentrations. D) – F) CMC calculations of the biosurfactants HAA (D), mono-rhamnolipid (E), di-rhamnolipid (F). The dotted lines (A) – C)) mark the calculated CMCs. The 5 mg/L surface tension values were not used to calculate the CMC as they are far away from the regression line (D)- F)). The order of the CMC (low to high) is not affected by this procedure. Error bars in all diagrams show the deviation from the mean of three individual experiments.

The calculations for CMC were made by plotting the measured surface tension values over the logarithmic concentration of the biosurfactant and splitting the graph in two parts. The split point was chosen to minimize the slope of the regression line used to fit the high concentration values (i.e., resulting in a line parallel to the x-axis).

## Comparison to *P. aeruginosa*

The carbon yield achieved with wild-type producer *P. aeruginosa* is significantly lower following Müller *et al*. [9]. At 7% [Cmol_RL_/Cmol_Glc_], it is less than 10% of the maximal theoretical yield. In addition, the rates are lower in the native producer (27 mg_RL_/(g_CDW_ h) opposed to 31 mg_RL_/(g_CDW_ h) reached with the recombinant cell factory). As rhamnolipid production in fermentations using the wild type can be maintained for 90 hours the product concentration is much higher (>100 g/L [10]) (see also Table S-4).

Table S-4. Fermentation characteristics of the three engineered recombinant biosurfactant producers.

| Organism | Substrate^1^ | Cell Dry Weight | Maximal Titer | Yield | Carbon Yield^2^ | Production Time^3^ | Specific RL-Production Rate^4^ | Reference |
| --- | --- | --- | --- | --- | --- | --- | --- | --- |
|  |  |  |  | [g_rhamnolipid_/ g_substrate_] | [Cmol_rhamnolipid_/ Cmol_substrate_] |  |  |  |
|  | [g/L] | [g_CDW_/L] | [g_RL_/L] |  |  | [h] | [g/(g_CDW_ h)] |  |
| *P. putida* KT2440 pPS05 | 12 | 3.4 | 2.40 | 0.23 | 0.35 (49%) | 23 | 0.031 | [4] |
| *P. putida* KT2440 pWJ02 | 10 | 1.8 | 3.26 | 0.33 | 0.49 (68%) | 48 | 0.038 | This work |
| *P. putida* KT2440 pSB01 | 10 | 5.7 | 1.54 | 0.15 | 0.27 (41%) | 22 | 0.012 | This work |
| *P. aeruginosa* PA01 | 250 | 16.3 | 39 | 0.16 | 0.07 (7%) | 90 | 0.027 | [9] |

^1^) For the recombinant strains glucose was used, while sunflower oil was the carbon source for *P. aeruginosa*.

^2^) For the calculation of yields during production on complex media, rhamnolipids and HAAs were assumed to be synthesized from the used carbon source, while media compounds were utilized for cell growth. The numbers in parenthesis show the percentage of the maximal possible theoretical yield reached.

^3^) The production time is the time when the maximal titer was reached.

^4^) The specific production rates were calculated as average over the fermentation time until the peak point was reached.

# References

1. Bagdasarian M, Lurz R, Ruckert B, Franklin FCH, Bagdasarian MM, Frey J, et al. Specific-purpose plasmid cloning vectors II. Broad host range, high copy number, RSF1010-derived vectors, and a host-vector system for gene cloning in *Pseudomonas.* Gene. 1981;16:237-47.

2. Panke S, Witholt B, Schmid A, Wubbolts MG. Towards a biocatalyst for (*S*)-styrene oxide production: Characterization of the styrene degradation pathway of *Pseudomonas* sp. strain VLB120*.* Appl Environ Microbiol. 1998;64:2032-43.

3. Hanahan D. Studies on transformation of *Escherichia coli* with plasmids*.* J Mol Biol. 1983;166:557-80.

4. Tiso T, Sabelhaus P, Behrens B, Wittgens A, Rosenau F, Hayen H, et al. Creating metabolic demand as an engineering strategy in *Pseudomonas putida* – Rhamnolipid synthesis as an example*.* Metab Eng Commun. 2016;3:234-44.

5. Wittgens A, Konstruktion neuer Produktionsstämme für die heterologe Rhamnolipidsynthese in dem nicht-pathogenen Wirt *Pseudomonas putida* KT2440. PhD Thesis*.* 2013, Ulm University.

6. Kovach ME, Elzer PH, Hill DS, Robertson GT, Farris MA, Roop II RM, et al. Four new derivatives of the broad-host-range cloning vector pBBR1MCS, carrying different antibiotic-resistance cassettes*.* Gene. 1995;166:175-6.

7. Silva-Rocha R, Martinez-Garcia E, Calles B, Chavarria M, Arce-Rodriguez A, de las Heras A, et al. The Standard European Vector Architecture (SEVA): a coherent platform for the analysis and deployment of complex prokaryotic phenotypes*.* Nucleic Acids Res. 2013;41:D666-D75.

8. Monteiro SA, Sassaki GL, de Souza LM, Meira JA, de Araujo JM, Mitchell DA, et al. Molecular and structural characterization of the biosurfactant produced by *Pseudomonas aeruginosa* DAUPE 614*.* Chem Phys Lipids. 2007;147:1-13.

9. Müller MM, Hörmann B, Syldatk C, Hausmann R. *Pseudomonas aeruginosa* PAO1 as a model for rhamnolipid production in bioreactor systems*.* Appl Microbiol Biotechnol. 2010;87:167-74.

10. Giani C, Wullbrandt D, Rothert R, Meiwes J, *Pseudomonas aeruginosa and its use in a process for the biotechnological preparation of L-rhamnose* (1997). Hoechst Aktiengesellschaft,
